# Modeling of Mixing‐Precipitation Processes: Agglomeration

**DOI:** 10.1002/ceat.201900551

**Published:** 2020-01-20

**Authors:** Pawel M. Orlewski, Marco Mazzotti

**Affiliations:** ^1^ ETH Zurich Institute of Process Engineering Sonneggstrasse 3 8092 Zurich Switzerland

**Keywords:** Agglomeration, Mixing‐precipitation process, Population balance equation, Reactive precipitation

## Abstract

A comprehensive description of the barium sulfate precipitation process in a wide range of supersaturations is presented. By using an additive to stabilize the particles, the decoupling of the primary from the secondary processes, as well as the agglomeration from aggregation was possible. By being able to study the two processes independently, a model describing the agglomeration of barium sulfate in the range of high supersaturations was validated experimentally for the first time. The proposed model has proven to describe the experiments with a high degree of accuracy in the whole range of supersaturations investigated. Additionally, by comparing agglomeration kernels of various complexity, ranges where simplifications are possible were identified, thus enabling the future development of models with better performance.

## Graphical abstract

By applying a rigorous approach, a comprehensive description of the barium sulfate precipitation process in a wide range of supersaturations was achieved. Using carefully designed experiments, a model describing the process was validated. The proposed model has proven to express the experiments with a high degree of accuracy in the whole range of supersaturations investigated.

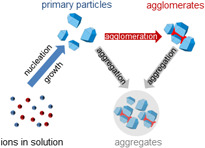

## 1 Introduction

Microparticle formation by precipitation is an important complex process, where process design, being challenging and expensive, may benefit from a model‐based approach. However, the development of such models for reactive precipitation processes is also challenging due to the very large driving force reached.

The precipitation of microparticles has been studied by various authors experimentally as well as theoretically using barium sulfate as a model compound. A lot of effort has been devoted to the decoupling of the different phenomena involved to study them separately, thus resulting in most of the studies focusing on a relatively small range of concentrations. This allowed for the identification of the governing mechanisms and for the development of the first principle equations describing the various mechanisms in the process like nucleation and growth 1, 2.

However, the industrial processes are operated under conditions where such decoupling is not possible and the various simplifications, like disregarding agglomeration, are no longer valid. Some work has been performed in studying agglomeration under such conditions, but the resulting models were only developed for limited values of supersaturation 3, 4, whilst a comprehensive description valid in a wide range of operating conditions and enabling predictions and process design is still lacking.

The aim of this work is to come up with a comprehensive description of the barium sulfate precipitation process in a wide range of supersaturations by applying a rigorous approach to the description of the kinetics of precipitation as well as to the definition of the experimental protocol. Ultimately this will provide new insight into the study of reactive precipitation at high supersaturations.

## 2 Primary Processes Revisited

The mechanisms and kinetics of barium sulfate precipitation have been studied by multiple authors over the recent years, resulting in a plethora of expressions for describing the primary mechanisms in the process – ranging from purely empirical to more predictive ones derived from first principles. The nucleation and growth rates calculated using different models proposed in the literature exhibit major differences among them. This inconsistency could be attributed to the difficulty in obtaining accurate experimental data for estimating the model parameters. This difficulty stems from the high driving force attained during reactive precipitation and the resulting small time‐ and length‐scales of the process. But even when referring to the purely predictive models in the literature, different expressions can be found. Therefore, in order to understand the differences between various approaches and to choose the most appropriate model, a brief overview of the description of primary processes in the scope of barium sulfate precipitation is presented below.

### 2.1 Driving Force of Precipitation

The driving force in precipitation is the difference in the chemical potentials between a molecule in the solution, *μ*_s_, and in the crystal bulk, *μ*_c_, defined as:

(1) $$\begin{array}{c} \Delta \mu =\mu_{s}-\mu_{c} \end{array}$$

The chemical potential of a molecule in the crystal bulk is equal to the molecule's equilibrium potential *μ*_e_:

(2) $$\begin{array}{c} \mu_{c}=\mu_{e} \end{array}$$

whereas the chemical potential of a non‐dissociating molecule in the solution can be expressed as:

(3) $$\begin{array}{c} \mu_{s}=\mu_{e}+k_{B}T\ln\left(a/a_{e}\right) \end{array}$$

where *k*_B_ is the Boltzmann constant, *T* is the temperature, *a* and *a*_e_ are the actual and the equilibrium activities of the molecule, respectively.

In case of ionic compounds like barium sulfate, whose molecules in solution are dissociated into ions, Eq. (3) becomes:

(4) $$\begin{array}{c} \mu_{s}=\nu_{1}\mu_{s,1}+\nu_{2}\mu_{s,2}+\ldots +\nu_{n}\mu_{s,n} \end{array}$$

where *ν*_i_ is the number of *i*‐th ions in the molecule, and *μ*_s,i_ is its corresponding chemical potential.

In the case of barium sulfate, by substituting Eqs. (2)‐(4), Eq. (1) can be written as:

(5) $$\begin{array}{c} \Delta \mu =k_{B}T\ln\left(\frac{a_{Ba^{2+}}}{a_{e,Ba^{2+}}}\frac{a_{{\mathrm{SO}}_{4}^{2-}}}{a_{e,{\mathrm{SO}}_{4}^{2-}}}\right) \end{array}$$

The supersaturation ratio, *s*, is therefore defined as:

(6) $$\begin{array}{c} s=\frac{a_{Ba^{2+}}}{a_{e,Ba^{2+}}}\frac{a_{{\mathrm{SO}}_{4}^{2-}}}{a_{e,{\mathrm{SO}}_{4}^{2-}}} \end{array}$$

Expressing activities as a product of concentration and an activity coefficient, Eq. (6) can be further transformed into:

(7) $$\begin{array}{c} s=\frac{c_{Ba^{2+}}c_{{\mathrm{SO}}_{4}^{2-}}}{K_{\mathrm{SP}}}\gamma_{\pm }^{2} \end{array}$$

where *c*_i_ is the molar concentration of the *i*‐th ion, *K*_SP_ is the solubility product, and $\gamma_{\pm }$ is the mean ionic activity coefficient. A comprehensive comparison of different models used for the calculation of the activity coefficient has been presented earlier 1. In this work, $\gamma_{\pm }$ is calculated using the Pitzer model, taking into account the complex formation of barium sulfate 1.

Whilst *s* is the correct definition of the supersaturation ratio, in the literature usually a different expression for its description is used, namely:

(8) $$\begin{array}{c} S=\sqrt{\frac{c_{Ba^{2+}}c_{{\mathrm{SO}}_{4}^{2-}}}{K_{\mathrm{SP}}}}\gamma_{\pm } \end{array}$$

where *s* = *S* ^2^. In most of the cases, this thermodynamically incorrect definition does not affect the final results because its effect is compensated by the estimated values of other model parameters. In the following section, an example showing the importance of using Eq. (7) instead of Eq. (8) to describe the supersaturation ratio in the context of the definition of nucleation rate is presented.

### 2.2 Nucleation

According to the classical nucleation theory (CNT), an expression for the nucleation rate *R*_N_ has the following general form:

(9) $$\begin{array}{c} R_{N}=A\;\exp\left(-\frac{B}{\ln^{2}\tilde{S}}\right) \end{array}$$

where $\tilde{S}$ is the supersaturation (we will consider both $\tilde{S}=s$ and $\tilde{S}=S$).

**Figure 1:**
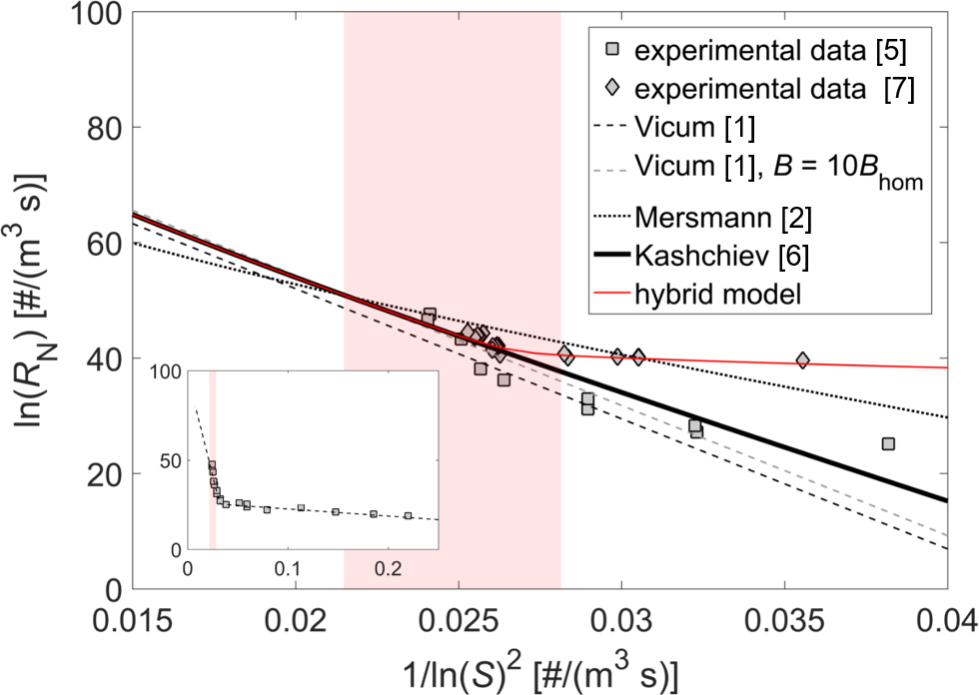
Comparison of the experimental (symbols) and theoretical (lines) homogeneous nucleation rates of barium sulfate as a function of supersaturation. Supersaturation was calculuted using Eq. (8). The red area corresponds to the range of supersaturation investigated in this work.

A common approach to obtain the kinetic parameters *A* and *B* is to linearize Eq. (9) and fit it to the experimental data. In Fig. 1, results for the homogeneous and heterogeneous nucleation obtained by Vicum et al. 1 (dashed line) using the experimental data by Nielsen 5 (square markers) and $\tilde{S}=S$ are presented. The measured nucleation rates in the homogeneous regime are in the range of 10^10^–10^20^ # m^−3^s^−1^.

Since the measurement of the nucleation rate is performed by counting under a microscope the number of crystals formed per unit volume, the resulting measured value must be, to some degree, fraught with an uncertainty. To get an idea of the effect that such uncertainty might have on the estimation of the kinetic parameters, the nucleation rates were recalculated using a value of the kinetic parameter *A*_hom_ ten times larger than that reported by Vicum et al. and the resulting values are also plotted in Fig. 1 (gray dashed line). As it can be readily seen, bearing in mind the accuracy of the experimental data in this range of supersaturation values, a change of an order of magnitude in *A*_hom_ yields a rather similar fitting of the experimental data. This observation leads to a drastic reduction of the confidence in the parameters estimated by fitting the experimental data.

Alternatively, especially in the range of high supersaturation, instead of relying on experiments to get the nucleation kinetics, a predictive model could be used. To describe the homogeneous nucleation rate in a fully predictive manner, the kinetic parameters *A*_hom_ and *B*_hom_ can be calculated based on the CNT and the nucleation theorem. In this approach, *B*_hom_ for the homogeneous nucleation can be expressed as 2, 6:

(10) $$\begin{array}{c} B_{\mathrm{hom}}=\frac{16}{3}\pi V_{0}^{2}{\left(\frac{\gamma }{k_{B}T}\right)}^{3} \end{array}$$

where *V*_0_ is the molecular volume given by *M*_c_/*ρ*_c_ *N*_A_, *k* is the Boltzmann constant, *T* is the temperature, and the specific surface energy of the cluster/solution interface, *γ*, can be defined as:

(11) $$\begin{array}{c} \gamma =0.514k_{B}{\mathrm{TV}}_{0}^{-2/3}\ln\left(\frac{1}{V_{0}C_{e}}\right) \end{array}$$

where *C*_e_ is the molecular solubility defined as $\sqrt{K_{\mathrm{SP}}}N_{A}$.

In the scope of the barium sulfate precipitation, a common expression for *A*_hom_ (for homogeneous nucleation) is that proposed by Mersmann et al. 2:

(12) $$\begin{array}{c} A_{\mathrm{hom}}=\frac{3}{2}{\left(\frac{\gamma }{k_{B}T}\right)}^{1/2}V_{0}D{\left(C_{e}\tilde{S}\right)}^{7/3} \end{array}$$

with $\tilde{S}=S$, and the monomer diffusion coefficient, *D*, being defined as:

(13) $$\begin{array}{c} D=\frac{k_{B}T}{3\pi \eta L_{m}} \end{array}$$

where *η* is the viscosity and *L*_m_ is the molecular diameter, defined as:

(14) $$\begin{array}{c} L_{m}={\left(\frac{6}{\pi }V_{0}\right)}^{1/3} \end{array}$$

In Fig. 1 (dotted line), the nucleation rates calculated using Eqs. (9)–(14) with $\tilde{S}=S$ are indicated. As it can be seen, they fit the experimental data rather poorly. This is often attributed to the difficulty in calculating the specific surface energy of the cluster/solution interface and is fixed by changing it to the value which results in a better match. But, as pointed out by Kashchiev and van Rosmalen 6, the reason for the poor fit lies more likely in the incorrect definition of the monomer attachment frequency and of the equilibrium concentration of nuclei (see 6 for more details). After applying the necessary changes, they report the following expression for *A*_hom_ 6:

(15) $$\begin{array}{c} A_{\mathrm{hom}}={\left(\frac{k_{B}T}{\gamma V_{0}^{2}}\right)}^{1/2}DC_{e}\ln\left(\tilde{S}\right)\tilde{S} \end{array}$$

with $\tilde{S}=s=S^{2}$. Nucleation rates calculated using Eqs. (9)–(11) and Eqs. (13)–(15), with the supersaturation in Eq. (9) and (15) defined using Eq. (7), are plotted in Fig. 1 (solid line). As it can be seen, the calculated values fit the experimental data very well. Moreover, since this expression is derived purely from theoretical considerations, apart from being free of the inaccuracy stemming from the quality of the experimental data used for parameter estimation, it is also applicable to supersaturation levels outside the range used by Nielsen 5, where in the case of fitted parameters an extrapolation would be needed.

At this point, the importance of employing an appropriate expression to describe the supersaturation ratio should be underlined. In the case of parameters *A* and *B* being independent of supersaturation, when fitting parameters using Eq. (9), it is irrelevant if supersaturation is defined as *S* ^2^ or *S*, because the exponent can be pulled out in front of the logarithm and lumped into the constant *B*. But, when using Eq. (15) to define *A* as a function of supersaturation, this is no longer possible and the importance of applying Eq. (7) is apparent.

In case of heterogeneous nucleation, a purely theoretical derivation of the expressions for the kinetic parameters is no longer possible because it would require an assumption about the shape of a heterogeneously formed cluster on the solid substrate. Therefore, in this work, the kinetic parameters *A*_het_ and *B*_het_ are defined as:

(16) $$\begin{array}{c} A_{\mathrm{het}}=aA_{\mathrm{hom}} \end{array}$$

(17) $$\begin{array}{c} B_{\mathrm{het}}=bB_{\mathrm{hom}} \end{array}$$

where *a* and *b* are the proportionality constants that need to be estimated from the experimental data.

Since the heterogeneous nucleation is very system‐dependent, instead of using the experimental data by Nielsen 5 which was obtained with a batch reactor, we have used the experimental data by Mohanty et al. 7 which was obtained using a continuous setup similar to the one used in this work. The parameters *a* and *b* were therefore estimated by fitting Eqs. (7), (9), (10), (15)–(17) to the experimental data in 7 and are equal to 3 × 10^−21^ and 0.001, respectively. In Fig. 1, the resulting nucleation rate calculated by a hybrid model, where the fully predictive homogeneous term (Eqs. (7), (9), (10), (15)) is combined with the estimated heterogeneous term (Eqs. (7), (9), (10), (15)–(17)), is plotted (red solid line) together with the experimental data obtained by Mohanty et al. 7 (lozenge markers).

### 2.3 Growth

The growth of crystals is usually described as a two‐step process, where the solute molecules first diffuse from the bulk to the crystal surface (diffusion step) after which they are integrated into the crystal lattice (integration step). Depending on the supersaturation, this process can be either integration‐ or diffusion‐limited. Due to the high supersaturations reached during reactive precipitation processes, the growth rate of barium sulfate is usually assumed to be diffusion‐limited throughout the whole process 4, 8, 9, also when the supersaturation is almost completely depleted as the growth of crystals at low supersaturations is negligible compared to that at high supersaturations. However, in this work, this assumption is not valid as, alongside the primary processes, we are also interested in describing agglomeration, which represents a considerable contribution also at low supersaturations, thus requiring the use of a two‐step model that accounts for both phenomena.

The integration step can be described as 1, 10:

(18) $$\begin{array}{c} G=k_{r}{\left({\tilde{S}}_{\mathrm{int}}-1\right)}^{2} \end{array}$$

where *k*_r_ is the integration rate coefficient and the subscript “int” refers to the crystal‐solution interface.

In this case whether the supersaturation is defined as *S* ^2^ or *S* is not as crucial as in the case of nucleation (see Sect. 2.2). The reason for this is that the above given power law expression is actually an approximation of the growth mechanisms governing the integration of molecules in the crystal lattice, which have a general form of $G\sim \ln\left({\tilde{S}}_{\mathrm{int}}\right)$ 11. Therefore, the exponent can be pulled out in front of the logarithm and lumped into the constant. In this work, $\tilde{S}=S$ and the value of *k*_r_ = 9.1 × 10^−12^ m s^−1^ obtained by Vicum et al. 1 using the data gathered by Nielsen and Toft 10 have been used.

The diffusion step can be described as 1, 11:

(19) $$\begin{array}{cc} G=k_{D}\left(a_{i}-a_{i,\mathrm{int}}\right), & i=Ba^{2+},{\mathrm{SO}}_{4}^{2-},\mathrm{BaS}O_{4} \end{array}$$

where *k*_D_ is the activity‐based mass transfer coefficient.

Obtaining reliable experimental values of growth rate in the range of high supersaturation is difficult not only due to the small time‐ and length‐scales of the process, but also because nucleation and growth cannot be decoupled. Therefore, due to the scarcity of experimentally measured growth rates at high supersaturations, usually a constant value of *k*_D_ is used 1, 12, 13 with the value chosen from the experimentally obtained range of 10^−5^–10^−4^ m kg s^−1^mol^−1^ 12, 14. Alternatively, *k*_D_ can be calculated using an expression derived from the mass transfer equation:

(20) $$\begin{array}{c} k_{D}=\frac{\mathrm{ShD}M_{c}\;\rho_{s}}{L\;\rho_{c}} \end{array}$$

where *L* is the diameter of a particle, *ρ*_s_ is the solution density, and *Sh* is the Sherwood number which, assuming that the particles are spheres smaller than 1 µm, is constant and equal to 2 15.

To check if this assumption holds also in the case of growth of particles with the size close to the critical nucleus, the size of molecule, *L*_m_, can be compared with the critical nucleus diameter, *L*_c_, defined as 2:

(21) $$\begin{array}{c} L_{c}=\frac{4\gamma V_{0}}{k_{B}T\ln\left(S^{2}\right)} \end{array}$$

In the range of supersaturations investigated in this work, *L*_c_ ≅ 2*L*_m_ = 10^−9^ m, thus indicating that the nucleus consists of only a couple of molecules and, consequently, that the assumption of sphericity might not be valid anymore. The experimental data for very small particles is scarce; however, some researchers have reported *Sh* much lower than 2 14, 16. If this discrepancy between the theoretical limit and the experimental results is due to the experimental error or to the effect of shape is unclear. However, for simplicity, in this work a constant value of *k*_D_ = 8 × 10^−5^ m kg s^−1^mol^−1^ is used.

By substituting Eq. (19) and (8), the activity at the crystal‐solution interface can be eliminated, and Eq. (18) can be written as:

(22) $$\begin{array}{c} G=k_{r}{\left(\sqrt{\left(a_{Ba^{2+}}-\frac{G}{k_{D}}\right)\left(a_{{\mathrm{SO}}_{4}^{2-}}-\frac{G}{k_{D}}\right)/K_{\mathrm{SP}}}-1\right)}^{2} \end{array}$$

## 3 Describing Agglomeration

Apart from the primary processes described in the previous section, particles in a suspension can also undergo secondary processes, namely, agglomeration, aggregation, and breakage. When particles collide, they can form ensembles that can be held together either by crystalline bridges (agglomerates) or by the van der Waals or electrostatic forces (aggregates). Due to the lack of a solid connection between the particles, the aggregates can be re‐dispersed by either changing the ionic strength of the solution or by changing the flow conditions or by introducing additives, thus making aggregation a reversible process. On the other hand, due to the much higher strength of the crystalline bridge, agglomeration is considered to be irreversible as the agglomerates can only be separated by applying high shear and by physically breaking them apart. Since the experimental procedure applied in this study allows for the decoupling of aggregation from agglomeration (see Sect. 5.1), only agglomeration is considered.

The rate constant of agglomeration, also referred to as the agglomeration kernel, is typically expressed as a product of the frequency of particle collisions and the probability of them forming a stable agglomerate, defined as:

(23) $$\begin{array}{c} \beta =\psi \beta_{\mathrm{col}} \end{array}$$

where *ψ* is the sticking probability and *β*_col_ is the collision kernel. Below, a brief summary of approaches to describe the two terms is presented.

### 3.1 Sticking Probability

Sticking probability is expressed as a function of a ratio between the cementation time *t*_c_ and the interaction time *t*_r_. Cementation time is the time needed to build a bridge strong enough to withstand the hydrodynamic forces acting on the ensemble of particles and to keep the two particles together, the interaction time between the two colliding particles is the time available to them to build such a bridge. In the literature, two expressions for the sticking probability are proposed 3:

(24) $$\begin{array}{c} \psi =\exp\left(-t_{c}/t_{r}\right) \end{array}$$

(25) $$\begin{array}{c} \psi ={\left(1+t_{c}/t_{r}\right)}^{-1} \end{array}$$

Eq. (24) is derived from the theory of drop coalescence, whereas Eq. (25) expresses proportionality of *ψ* to the growth rate. Several authors have reported a better agreement with the experimental data using Eq. (25) instead of Eq. (24) 3, 17. In this work, Eq. (25) is used to describe the sticking probability.

The cementation time can be derived from a force balance on the crystalline bridge and, assuming a point contact between the colliding particles, can be defined as 18:

(26) $$\begin{array}{c} t_{c}=k_{c}\sqrt{\rho_{c}\nu {\left(\varepsilon /\nu \right)}^{0.5}}\bar{L}/G \end{array}$$

where *k*_c_ is a constant combining parameters of hydrodynamic and material properties, *ε* is the energy dissipation rate, *ν* is the kinematic viscosity, and $\bar{L}$ is the mean diameter of the agglomerate.

The interaction time is assumed to be the time between two rupture events in the turbulent flow and can be estimated as the lifetime of small turbulent eddies. Several authors have proposed different length‐scales to characterize those small eddies – ranging from the Kolmogorov microscale 19, through Lagrangian microscale 3 to the largest, energy‐containing scale 20. Even though the resulting expressions for calculating the lifetime of the eddies are slightly different, they all yield similar results 21. In this work, the interaction time has been estimated by the lifetime of a turbulent eddy, defined as 20:

(27) $$\begin{array}{c} t_{r}=\frac{C_{\mu }^{0.75}}{\sqrt{2}}\frac{k}{\varepsilon } \end{array}$$

where *k* is the turbulent kinetic energy and *C*_µ_ = 0.09 is a model constant in the *k‐ε* model of turbulence.

### 3.2 Collision Kernel

Usually, two types of particle collisions are considered in the literature, namely, collisions due to Brownian diffusion (perikinetic) and collisions due to convective transport by fluid motion (orthokinetic). The orthokinetic collision kernel *β*_ortho_ can be written as 22:

(28) $$\begin{array}{c} \beta_{\mathrm{ortho}}=1.3{\left(\frac{\varepsilon }{\nu }\right)}^{0.5}{\left(\frac{L_{i}+L_{j}}{2}\right)}^{3} \end{array}$$

where *L*_i_ and *L*_j_ are the diameters of the two colliding particles.

The perikinetic collision kernel, *β*_peri_, can be defined as 23:

(29) $$\begin{array}{c} \beta_{\mathrm{peri}}=2\pi D_{\mathrm{ij}}^{\infty }\left(L_{i}+L_{j}\right)/W \end{array}$$

where *W* is the stability ratio. The mutual diffusion coefficient of colliding particles, $D_{\mathrm{ij}}^{\infty }$, is defined as

(30) $$\begin{array}{c} D_{\mathrm{ij}}^{\infty }=D_{i}+D_{j}=\frac{k_{B}T}{3\pi \eta }\left(\frac{1}{L_{i}}+\frac{1}{L_{j}}\right) \end{array}$$

In the original work by von Smoluchowski, the interparticle forces were neglected resulting in *W* = 1. When electrostatic forces are considered, *W* can be written as 24:

(31) $$\begin{array}{c} W=0.5\left(L_{i}+L_{j}\right)\int_{0}^{\infty }\frac{\exp\left(\varphi_{\mathrm{total}}\left(a\right)/k_{B}T\right)}{{\left(0.5\left(L_{i}+L_{j}\right)+a\right)}^{2}}da \end{array}$$

where *a* is the surface‐to‐surface distance between the colliding particles and *φ*_total_ is the total potential energy of the interparticle forces, which is a function of their distance. Based on the DLVO theory (after Derjaguin, Landau, Verwey, Overbeek), *φ*_total_ can be calculated as the sum of the attractive van der Waals contribution, *φ*_vdW_, and of the repulsive electrostatic contribution, *φ*_el_:

(32) $$\begin{array}{c} \varphi_{\mathrm{total}}\left(a\right)=\varphi_{\mathrm{vdW}}\left(a\right)+\varphi_{\mathrm{el}}\left(a\right) \end{array}$$

The van der Waals contribution can be calculated using the expression proposed by Hamaker 25:

(33) $$\begin{array}{c} \varphi_{\mathrm{vdW}}\left(a\right)=-\frac{A}{6}\left(\begin{array}{c} \frac{0.5L_{i}L_{j}}{a^{2}+\left(L_{i}+L_{j}\right)a}+\frac{0.5L_{i}L_{j}}{a^{2}+\left(L_{i}+L_{j}\right)a+L_{i}L_{j}}\\ +\ln\left(\frac{a^{2}+\left(L_{i}+L_{j}\right)a}{a^{2}+\left(L_{i}+L_{j}\right)a+L_{i}L_{j}}\right) \end{array}\right) \end{array}$$

where *A* is the Hamaker constant, which is 1.7 × 10^−20^ J for barium sulfate in water 26.

The electrostatic repulsion of particles in a solution is mainly due to the adsorption of ions on the particle surface and therefore depends not only on the size and distance of the particles, but also on the concentration of the ions in solution. Due to the charge on the surface resulting from the adsorbed ions, particles are surrounded by ions of an opposite sign forming an electric double‐layer. When two particles with the same surface potential approach each other, their double‐layers overlap causing a repulsive force. The electrostatic potential energy of such interaction can be written as 27:

(34) $$\begin{array}{c} \begin{array}{c} \varphi_{\mathrm{el}}\left(a\right)=64\pi \varepsilon_{r}\varepsilon_{0}{\left(\frac{k_{B}T}{e}\right)}^{2}\tanh^{2}\left(\frac{\mathrm{ze}\psi_{0}}{4k_{B}T}\right)\\ \left(\frac{L_{i}L_{j}}{2(L_{i}+L_{j}+2a)}\right)\exp\left(-\kappa a\right) \end{array} \end{array}$$

where *e* is the elementary electric charge, *z* is the valence, *ε*_r_ is the relative permittivity, *ε*_0_ is the electric field constant, *Ψ*_0_ is the surface potential, and *κ* is the Debye length defined as:

(35) $$\begin{array}{c} \kappa ={\left(\frac{2e^{2}N_{A}I}{\varepsilon_{r}\varepsilon_{0}k_{B}T}\right)}^{0.5} \end{array}$$

*I* is the ionic strength of the solution.

The main difficulty in applying Eq. (34) to calculate the electrostatic potential energy is in obtaining the correct values for the surface potential. Often, *Ψ*_0_ is calculated using a measured *ζ*‐potential. This approach is feasible not only when the experiments have a rather high accuracy, but also when the relation of the *ζ*‐potential to the surface potential is known accurately. Unfortunately, this is not always the case and usually the *ζ*‐potential and the surface potential are just assumed to be equal 28.

Alternatively, one could calculate the surface potential from the surface charge density obtained using the Grahame equation 27. Unfortunately, in order to do so, the adsorption isotherms of all the ions adsorbing on the surface need to be known and this data is not always available. Luckily, in the case of a reactive precipitation of barium sulfate using barium chloride and sodium sulfate as reactants, it has been shown that the concentration of sulfate, sodium, and chloride ions have a negligible effect on the *ζ*‐potential as compared to the barium ions 29. Therefore, it can be assumed that the barium ions are the only potential determining ions and the overall surface potential can be calculated using the well‐known Nernst equation:

(36) $$\begin{array}{c} \psi_{0}=\frac{k_{B}T}{2e}\ln\left(\frac{c_{Ba^{2+}}}{c_{{\mathrm{Ba}}_{\mathrm{iso}}^{2+}}}\right) \end{array}$$

where $c_{{\mathrm{Ba}}_{\mathrm{iso}}^{2+}}$ is the concentration of the barium ions at the isoelectric point.

Moreover, the effect of ions on the relative permittivity needs to be considered. Gavish et al. 30 have studied the effect of NaCl concentration on the relative permittivity of barium sulfate and stated that in the range investigated in this work such effect is less than 3 % and, therefore, can be considered negligible.

When two particles are approaching each other in the solution, apart from the electrostatic forces, there are also hydrodynamic forces present resulting from an additional resistance caused by the necessary squeezing of the liquid between the two solid bodies. To include the viscous interactions as well, Spielman 31 proposed a further modification of Eq. (31) by introducing an additional parameter accounting for the change in the diffusion coefficient as particles approach:

(37) $$\begin{array}{c} W=0.5\left(L_{i}+L_{j}\right)\int_{0}^{\infty }\frac{\exp\left(\varphi_{\mathrm{total}}\left(a\right)/k_{B}T\right)}{D*\left(a,L_{i}/L_{j}\right){\left(0.5\left(L_{i}+L_{j}\right)+a\right)}^{2}}da \end{array}$$

where $D*\left(a,L_{i}/L_{j}\right)$ is the ratio between the actual diffusion coefficient, *D*_ij_, taking into account the distance between the particles and their size, and the diffusion coefficient calculated using Eq. (30) assuming that particles are widely separated relative to their dimensions, i.e., $D*\left(a,L_{i}/L_{j}\right)=D_{\mathrm{ij}}/D_{\mathrm{ij}}^{\infty }$.

**Figure 2:**
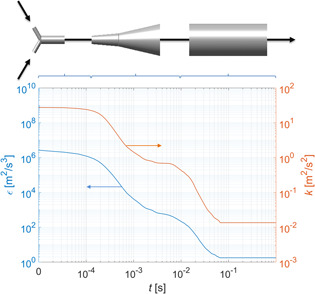
Evolution of the energy dissipation rate (blue line, left axis) and the turbulent kinetic energy (red line, right axis) along the reactor path as a function of the residence time in the reactor.

In this work, results for a set of *a* and of *L*_i_/*L*_j_ reported in the literature (see Fig. 2 in 31) were used to create a look‐up table that was used for interpolating the required values of D* during our calculations.

Therefore, by using Eqs. (28)–(30) and (31)–(37) and by assuming that the peri‐ and orthokinetic regimes are independent, hence, their contributions can be simply added to each other, the overall collision kernel can be calculated:

(38) $$\begin{array}{c} \beta_{\mathrm{col}}=\beta_{\mathrm{peri}}+\beta_{\mathrm{ortho}} \end{array}$$

Baldyga and Orciuch 32 have proposed an expression for an agglomeration kernel when the flux additivity cannot be applied. However, it has been shown that in the case of fast agglomerating systems, both contributions are independent 33 and especially for the case of barium sulfate agglomeration, both models yield similar results 3. Therefore, in this work Eq. (38) is applied.

## 4 Modeling

### 4.1 Population Balance Model

To model the precipitation of barium sulfate, a population balance equation (PBE) is used. Assuming a perfectly mixed system, the PBE using the length, *L*, as internal coordinate can be written as:

(39) $$\begin{array}{c} \frac{\partial n}{\partial t}=-G\frac{\partial n}{\partial L}+B-D \end{array}$$

where *n* is the number density of particles, *t* is the residence time given by the axial coordinate along the tubular reactor divided by the velocity of the solution, and *B* and *D* are the birth and death terms due to agglomeration.

The PBE is coupled with a mass balance of the solute in the liquid phase, which, for a perfectly mixed system, is expressed as:

(40) $$\begin{array}{c} \frac{\partial c}{\partial t}=-\frac{\pi }{2}\frac{\rho_{c}}{M_{c}}G\int_{L_{c}}^{\infty }n\left(L,t\right)L^{2}dL-\frac{\pi }{6}\frac{\rho_{c}}{M_{c}}R_{N}L_{c}^{3} \end{array}$$

To complete the model, the following boundary and initial conditions are used:

(41) $$\begin{array}{c} n\left(L_{c},t\right)=R_{N}/G \end{array}$$

(42) $$\begin{array}{c} n\left(L,0\right)=0 \end{array}$$

(43) $$\begin{array}{c} c\left(0\right)=c_{0} \end{array}$$

To solve the PBE, the method of characteristics with moving pivot was applied 34 and the resulting set of ordinary differential equations (ODEs) were solved using the MATLAB ode45 solver. As an average particle size, *L*_43_ was chosen:

(44) $$\begin{array}{c} L_{43}=\frac{m_{4}/m_{3}}{{\left(\pi /k_{a}\right)}^{1/3}} \end{array}$$

where *m*_3_ and *m*_4_ are the third and fourth moment of the distribution, respectively.

### 4.2 Computational Fluid Dynamics (CFD)

The agglomeration kernel depends, among others, on the fluid dynamics in the system. Due to the series of expansions, the flow conditions in the mixer change as the solution moves from the inlets through the mixing zone and out to the reaction tube (see Fig. 2). Therefore, even though the mixing effects can be neglected, the evolution of the energy dissipation rate and of the turbulent kinetic energy along the reactor path needs to be known. To obtain the required values of *ε* and *k*, a CFD model of the system has been developed using the commercial CFD software Fluent 17.2. To solve the Navier‐Stokes equations, a standard RNG *k–ε* model of turbulence has been used together with an enhanced wall model to describe the near‐wall flow.

The average values of *ε* and *k* along the reactor were calculated using the following expressions:

(45) $$\begin{array}{c} \bar{\varepsilon }\left(x\right)=\int_{0}^{\infty }\varepsilon \left(x\right)f\left(\varepsilon \left(x\right)\right)d\varepsilon \end{array}$$

(46) $$\begin{array}{c} \bar{k}\left(x\right)=\int_{0}^{\infty }k\left(x\right)f\left(k\left(x\right)\right)dk \end{array}$$

where *x* is the position along the length of the tube; *f*(*ε*(*x*)) and *f*(*k*(*x*)) are the position‐dependent probability distribution function of the energy dissipation and the turbulent kinetic energy in the reactor, respectively. The resulting dependence, expressed as a function of residence time, is illustrated in Fig. 2.

## 5 Experimental

### 5.1 Chemicals and Methods

The chemicals barium chloride dihydrate (≥ 99 %, Sigma‐Aldrich Switzerland) and sodium sulfate (≥ 99 %, Sigma‐Aldrich Switzerland) were used in all experiments. Precipitation of barium sulfate was carried out by mixing aqueous solutions of barium chloride and sodium sulfate under stoichiometric conditions and at a constant temperature of 25 °C. First, stock solutions were prepared by dissolving the salts in deionized water. Afterwards, due to the very small time‐scale of the process requiring rapid mixing, reactants were continuously mixed in a Y‐mixer (inlet diameter 0.5 mm, outlet diameter 1 mm, angle between inlets 120°) at an equal mass flow rate of 20 kg h^−1^, thus ensuring that mixing does not influence the precipitation kinetics 8.

A continuous, pulsation‐free flow has been achieved by means of a set of micro‐gear pumps (mzr‐11508X1, HNP Germany) coupled with a Coriolis mass flow meter and controller (M15, Bronkhorst, The Netherlands). After mixing, the solution entered a tubular reactor with a diameter of 4 mm and length of 18 m, resulting in a residence time of about 36 s – sufficient to completely deplete supersaturation. After leaving the reactor, a sample of 100 mL was collected. After diluting the sample tenfold, the size of particles was measured by dynamic light scattering (DLS; Zetasizer Nano ZS, Malvern Instruments, UK).

### 5.2 Stabilization

During barium sulfate precipitation various primary and secondary processes take place simultaneously. In order to study the effect of agglomeration on the final product's properties, first the primary mechanisms needed to be decoupled from the secondary mechanisms to obtain a reference case where no agglomeration takes place. Second, agglomeration had to be distinguished from aggregation. Agglomeration and aggregation of barium sulfate can be suppressed either by electrostatic or by steric stabilization. In case of the electrostatic stabilization on the one hand, precipitation is performed with an excess of barium ions $\left(c_{Ba^{2+}}/c_{{\mathrm{SO}}_{4}^{2-}}>1\right)$, which adsorb on the crystal's surface, thus increasing the repulsive forces and therefore reducing the collision rate of particles 35. On the other hand, in the case of the steric stabilization, a polymer adsorbs at the surface, thus physically separating particles and subsequently reducing the sticking probability.

The problem with the electrostatic stabilization is that even though the primary processes can be decoupled from the secondary ones, the decoupling of agglomeration from aggregation is not possible as it would require the introduction of barium ions into the system after the supersaturation is depleted. This would lead to an increase of supersaturation and reintroduction of precipitation. For more information on the non‐stoichiometric precipitation of barium sulfate, see 1. Therefore, in this work steric stabilization using as a surfactant an aqueous solution of a polyethercarboxylate (Melpers45, BASF, Germany) has been applied.

Two types of experiments with the surfactant were performed: (1) to study the primary processes only, the surfactant was added to the barium chloride solution, thus preventing both agglomeration and aggregation right from the beginning of the crystallization process; (2) to decouple agglomeration from aggregation, the surfactant was added to the sample collected at the end of the reaction tube where the supersaturation was already depleted, thus resulting in the de‐aggregation of crystals.

## 6 Results

Two types of experiments have been performed: one where only primary processes were active and one where growth, nucleation, and agglomeration took place simultaneously. Both cases were investigated for a wide range of initial salt concentrations varying from 0.015 to 0.1 mol kg^−1^. In Tab. 1, all the initial concentrations studied together with the corresponding supersaturations are reported. To make easier the comparison with the previously reported work on the barium sulfate precipitation, where the quantity *S* defined by Eq. (8) was used, here also the same definition of the supersaturation was employed.

**Table 1:** Summary of all the initial concentrations of reagents used in the experiments with the corresponding supersaturation calculated using Eq. (8).

$c_{Ba^{2+}}$	$c_{{\mathrm{SO}}_{4}^{2-}}$	S
0.015	0.015	404
0.020	0.020	473
0.025	0.025	532
0.030	0.030	583
0.035	0.035	629
0.050	0.050	741
0.100	0.100	978

### 6.1 Particle Stabilization

To determine the amount of additive required to stabilize the particles under the investigated conditions, experiments were performed where the initial concentration of additive in the inlet, *c*_add_, was varied from 0 to 16 g kg^−1^ (concentration of polymer per mass of solution). We have observed that for all the supersaturations investigated, *c*_add_ = 4.5 g kg^−1^ was sufficient to see no more change in the measured particle size, which is in agreement with the data reported in the literature 8, 36, 37. In the next section (Sect. 6.2), it will be discussed whether agglomeration was completely suppressed or not.

### 6.2 Primary Processes

**Figure 3:**
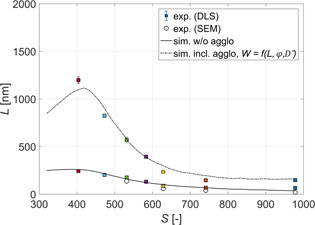
Comparison between experimental (symbols) and simulation (lines) results for primary particles and agglomerates. Supersaturation was calculated using Eq. (8).

To see the effect of agglomeration on the final size of particles, first the experiments where only nucleation and growth are active were performed. In Fig. 3, the measured average sizes of crystals obtained using various initial supersaturations and the additive in the barium chloride feed solution are depicted. As expected, by increasing the initial supersaturation, the size of crystals decreases.

**Figure 4:**
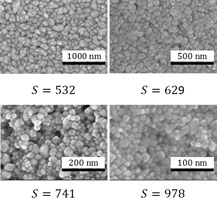
SEM pictures of barium sulfate crystals obtained at different supersaturations. Supersaturation was calculated using Eq. (8).

To check the accuracy of the DLS measurements, scanning electron microscopy (SEM) pictures of crystals obtained from experiments performed at four different supersaturations, namely, 532, 629, 741, and 978 (calculated using Eq. (8)), have been taken (see Fig. 4). By measuring the sizes of individual crystals in each picture, an average size of crystals for each of the investigated supersaturations was obtained and the results are also plotted in Fig. 3 (circles).

As it can be seen, the results obtained from the SEM match very well with the DLS measurements, thus indicating that the DLS measures the size of crystals accurately enough. Additionally, because the measured size corresponds to the size of the primary particles, the amount of additive used was indeed sufficient to successfully suppress the secondary mechanisms. The slight difference in the case of the highest supersaturation could be explained by the fact that primary crystals are too small for the additive particle to attach to their surface and only when they have agglomerated to a larger size, the stabilization was effective.

In Fig. 3, the simulation results are also indicated (solid line). As it can readily be seen, the simulations match the experiments rather well. The good agreement with the experimental data is especially remarkable in the range when homogeneous nucleation occurs (*S* > 450) where the model is almost fully predictive with only one parameter obtained from experiments.

Given the abundance of barium sulfate precipitation studies, one could try and compare the experimental as well as the simulation results obtained in this work with those reported in the literature. Unfortunately, it is not as trivial as it may seem at first sight. Schwarzer et al. have used a different system where the surface potential can no longer be approximated by Eq. (36) and the additional effect of adsorption of protons must be considered 9, 38. Vicum et al. have employed the same system as the one in this work allowing for a comparison between simulation results, but they have studied the precipitation process in a continuous stirred‐tank reactor (CSTR) where the assumption of a perfectly mixed system is no longer valid and the effects of mixing have to be considered 1, 39. Furthermore, the experiments were performed at much lower supersaturations where the shape of precipitated barium sulfate crystals differ greatly from a sphere, thus requiring the use of different shape factors. Nevertheless, their model can be compared with the approach in this work.

**Figure 5:**
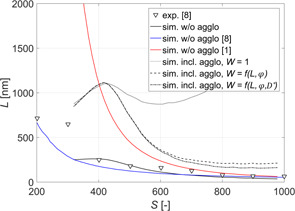
Solid lines correspond to the simulation results for the description of primary particles using different models: the model used in this work (black line) and the models proposed in the literature (blue and red lines). Dashed lines correspond to the simulation results for the description of agglomerates using different expression for stability ratio. Supersaturation was calculated using Eq. (8). Experimental results (symbols) are taken from 9.

From the available literature, only the results reported by Kügler et al. 8 can be taken for comparison, because they used the same system and a very similar experimental setup. In Fig. 5, the experimental (triangles) as well as simulation (blue line) results obtained by Kügler et al. are compared with the model used in this work (black solid line). The model used by Kügler et al. was proposed by Mersmann et al. 2 and has been described in Sect. 2.2. As it can be seen, the experimental results match closely with those reported in this work. Moreover, in the range of supersaturation between 600 and 1000, both models perform similarly. But for lower supersaturations (*S* ∈(400,600)) the model used in this work seems to describe the experimental results better than the one proposed by Mersmann et al.

The lowest supersaturation investigated by Kügler et al. has a value of about 200 whereas in this work, due to the expected change of shape of precipitated crystals, the lowest simulated supersaturation value is 350. As reported by Podgórska 1, 40, for supersaturation values lower than 350, precipitated crystals have a surface shape factor equal to 348, i.e., roughly ten times larger than the one for higher supersaturations, thus resulting in much higher actual growth rates and an underestimation of crystal sizes when not taking it into an account. This could explain the sudden increase in the size of the precipitated crystals in the case of supersaturation lower than 350 and the mismatch between the model presented in this work.

In Fig. 5, the simulation results using the empirical expressions proposed by Vicum et al. 1 (red line) are also displayed. For *S* > 800, the simulation results for all three models are very similar. For lower supersaturations though, the empirical model proposed by Vicum et al. differs from the one used in this work. The difference can be mainly attributed to the difference between the calculated nucleation rates. As it can be seen in Fig. 1, the difference between the nucleation rates calculated using the two approaches is increasing as the supersaturation decreases, thus highlighting the above‐mentioned inaccuracy in fitting nucleation rates at high supersaturations (see Sect. 2.2) and how a seemingly small change in the nucleation rate can result in a very different simulation result.

### 6.3 Agglomeration

In Fig. 3, the measured average sizes of agglomerates obtained using various initial supersaturations are indicated. To make sure that only agglomerates and not aggregates are measured, in those experiments the surfactant was added to the sample collected after the depletion of supersaturation, ergo when nucleation, growth, and agglomeration no longer took place. As it can be seen, for all supersaturations investigated, the measured size is larger than the size of primary particles. For higher supersaturations (*S* > 600) this difference is noticeably smaller as compared to the lower supersaturations (*S* < 600) where it is not only larger, but it also rises more rapidly with the increase of the size of primary particles. This difference could be attributed to the change of the agglomeration mechanism from perikinetic to orthokinetic as the size of particles increases. For smaller particles, the perikinetic mechanism is dominant where the agglomeration kernel linearly depends on the size of particles (see Eq. (29)). As the particles are becoming larger, the orthokinetic mechanism becomes dominant where the agglomeration kernel's dependence on the size of colliding particles is cubic (see Eq. (28)).

The effect of different agglomeration mechanisms can be further seen by looking into the simulation results. In Fig. 5, the simulation results using the model presented in Sect. 3 are also illustrated (dotted, dashed, and dotted‐dashed lines). The only model parameter, *k*_c_, has been estimated by fitting the model to the experimental data. As mentioned above (see Sect. 3), three different expressions for calculating the stability ratio were used with *k*_c_ = 7.8 × 10^−6^ when interparticle forces were neglected (*W* = 1), and *k*_c_ = 5 × 10^−6^ when electrostatic forces or electrostatic and hydrodynamic forces were considered (Eqs. (31) and (37), respectively).

As it can be seen in Fig. 5, for the largest primary particles (*S* < 400) all three approaches yield very similar results. This is expected, as for the largest particles, as already mentioned, agglomeration is mainly due to the orthokinetic mechanism which is not affected by the interparticle forces and depends only on the size of the particles and on the flow properties (see Eqs. (28) and (29)). As the size of the particles decreases and the perikinetic contribution becomes significant, only the models including the electrostatic interparticle forces are able to describe the experimental data well (dashed and dotted‐dashed lines in Fig. 5 obtained using Eqs. (31) and (37)). The effect of viscous forces becomes significant only for the smallest particles (*S* > 550), requiring the use of the stability ratio proposed by Spielman (Fig. 5, dotted‐dashed line). Overall, as can be seen in Fig. 3, by accounting for the electrostatic as well as for the viscous forces, the model proposed in Sect. 3 is able to describe experimental data in the whole range of supersaturations investigated.

Even more than in the case of primary processes, comparison of the agglomeration results presented in this work with those found in the literature is rather difficult. Kügler et al. 8 and Jasińska et al. 3 have studied the agglomeration of barium sulfate but since the energy dissipation rate in both cases is unknown, the calculation of the sticking probability as well as the collision kernel is not possible. Marchisio et al. 4 have investigated a much lower supersaturation, where the shape of precipitated barium sulfate crystals can no longer be approximated by a sphere. Given the above‐mentioned difficulties, no comparison with the literature has been possible.

## 7 Conclusions

By applying a rigorous approach to describe the kinetics of precipitation and to design the experimental protocol, a comprehensive description of the barium sulfate precipitation process in a wide range of supersaturations was achieved. The proposed experimental method allowed not only for the decoupling of the primary from the secondary processes but, more importantly, also of the agglomeration from aggregation. By being able to study the two processes independently, a model describing the agglomeration of barium sulfate in the range of high supersaturations could be validated experimentally for the first time.

The proposed model has proven to describe the experiments with a high degree of accuracy in the whole range of supersaturations investigated. Additionally, by comparing agglomeration kernels of various complexity ranges, where simplifications are possible, were identified, allowing for the development of models with better performance in the future.

## Symbols used

*A* [m^−3^s^−1^]: kinetic parameter
*A* [J]: Hamaker constant
*a* [–]: proportionality constant Eq. (16)
*a* [m]: surface‐to‐surface distance between colliding particles
*a*_i_ [mol kg^−1^]: activity of ion *i*
*B* [–]: thermodynamic parameter
*b* [–]: proportionality constant Eq. (17)
*C*_e_ [kg^−1^]: molecular solubility
*C*_µ_ [–]: constant in the *k‐ε* model of turbulence
*c*_i_ [mol kg^−1^]: concentration of species *i*
*D* [m^2^s^−1^]: diffusion coefficient
*e* [C]: elementary electric charge
*G* [m s^−1^]: growth rate
*I* [mol kg^−1^]: ionic strength
*K*_SP_ [–]: thermodynamic solubility product
*k* [m^2^s^−2^]: turbulent kinetic energy
*k*_B_ [J K^−1^]: Boltzman constant
*k*_c_ [–]: constant Eq. (26)
*k*_D_ [m kg s^−1^mol^−1^]: activity‐based mass transfer coefficient
*k*_r_ [m s^−1^]: integration rate coefficient
*L* [m]: diameter
*M*_c_ [kg kmol^−1^]: molecular mass
*N*_A_ [mol^−1^]: Avogadro constant
*n* [m^−3^]: number density of particles
*R*_N_ [m^−3^s^−1^]: nucleation rate
*S* [–]: supersaturation ratio Eq. (8)
$\tilde{S}$ [–]: supersaturation ratio
*Sh* [–]: Sherwood number
*s* [–]: supersaturation ratio Eq. (7)
*T* [K]: temperature
*t* [s]: time
*W* [–]: stability ratio
*z* [–]: valence

**Greek letters**
*β* [m^−3^s^−1^]: agglomeration kernel
*γ* [J m^−2^]: specific surface energy of the cluster/solution interface
$\gamma_{\pm }$ [–]: mean ionic activity coefficient
*ε* [m^2^s^−3^]: energy dissipation rate
*ε*_0_ [C^2^J^−1^m^−1^]: electric field constant
*ε*_r_ [m^2^s^−3^]: relative permittivity
*η* [kg m^−1^s^−1^]: dynamic viscosity
*κ* [m^−1^]: Debye length
*μ* [J]: chemical potential
*ν* [m^2^s^−1^]: kinematic viscosity
*ρ*_c_ [kg m^−3^]: crystal density
*φ*_el_ [J]: electrostatic potential energy
*φ*_total_ [J]: total potential energy of the interparticle forces
*φ*_vdW_ [J]: van der Waals potential energy
*ψ* [–]: stability ratio
*Ψ*_0_ [J C^−1^]: surface potential

**Abbreviations**
CFD: computational fluid dynamics
CNT: classical nucleation theory
DLS: dynamic light scattering
PBE: population balance equation
